# Prognostic impact of the “Sekhar grading system for cranial Chordomas” in patients treated with pencil beam scanning proton therapy: an institutional analysis

**DOI:** 10.1186/s13014-020-01547-x

**Published:** 2020-05-06

**Authors:** Anna-Lena Hottinger, Beat Bojaxhiu, Frank Ahlhelm, Marc Walser, Barbara Bachtiary, Stefan Zepter, Tony Lomax, Alessia Pica, Damien C. Weber

**Affiliations:** 1 Center for Proton Therapy, Paul Scherrer Institute, 5232 PSI West, Villigen, Switzerland; 2 Neuroradiology Department, Kantonsspital Baden, Baden, Switzerland; 3 Department of Physics, ETH Zürich, Zürich, Switzerland; 4 Radiation Oncology Department, University Hospital of Bern, Bern, Switzerland; 5 Radiation Oncology Department, University Hospital of Zürich, Zürich, Switzerland

**Keywords:** Skull base chordoma, Proton therapy, Pencil beam scanning, Prognostic grading system

## Abstract

**Background:**

Skull base chordomas are rare and heterogeneously behaving tumors. Though still classified as benign they can grow rapidly, are locally aggressive, and have the potential to metastasize. To adapt the treatment to the specific needs of patients at higher risk of recurrence, a pre-proton therapy prognostic grading system would be useful. The aim of this retrospective analysis is to assess prognostic factors and the “Sekhar Grading System for Cranial Chordomas” (SGSCC) by evaluating the larger cohort of patients treated at our institution as to determine its reproducibility and ultimately to ensure more risk adapted local treatments for these challenging tumors.

**Methods:**

We analyzed 142 patients treated for skull base chordomas between 2004 and 2016. We focused the analysis on the 5 criteria proposed for the SGSCC (tumor size, number of anatomic regions and vessels involved, intradural invasion, as well as recurrence after prior treatment) and classified our patients according to their score (based on the above mentioned criteria) into three prognostic groups, low-risk, intermediate-risk and high-risk. The three groups were then analyzed in regards of local control, local recurrence-free survival and overall survival.

**Results:**

The median follow up was 52 months (range, 3–152). We observed 34 (24%) patients with a local recurrence, resulting in a local control of 75% at 5 years. Overall survival was 83% at 5 years, 12 (9%) patients had died due to local progression. When split into the three prognostic groups according to the SGSCC the observed local control was 90, 72 and 64% (*p = 0.07*) in the low-, intermediate- and high-risk group, respectively. A similar correlation was observed for local recurrence-free survival with 93, 89 and 66% (*p = 0.05*) and for overall survival with 89, 83 and 76% *(p = 0.65)* for the same prognostic groups.

**Conclusions:**

After splitting our patient cohort into the three SGSCC risk groups, we found a trend towards better outcome for those patients with lower as opposed to higher scores. These results suggest that this prognostic grading system published by Sekhar et al. could be integrated in the management decision-tree for patients with skull base chordoma.

## Background

Chordomas are very rare tumors with an incidence of 0.08–0.1/100000 per year, making up 1–4% of all bone tumors [1–3]. Their cells derive from remnants of the embryonal notochord, occurring most commonly in the sacral region (50–60%) followed by the skull base (25–30%) [3, 4]. Although they are slow growing and histologically considered low-grade tumors that rarely metastasize, they are locally aggressive with a high local recurrence rate, causing severe morbidity and even death due to the proximity to critical structures such as nerves or – as in the case of skull base chordomas (SBC) – the brain stem. This means that local treatment is key, with maximally safe resection followed by radiation treatment as the standard of care [5–8]. However, due to the location of these tumors optimal aggressive local treatment, be it with surgery or radiation therapy, is also challenging, resulting in suboptimal local control in a substantial number of patients. Aiming at treatment improvement, many recent studies evaluated the prognostic factors of patients with SBC, such as tumor size, location and extent of surgery, showing a correlation between these factors and local recurrence rates as well as survival [9–12]. Even though prognostic factors could be identified, only one comprehensive score had been developed implementing these factors in the pre-treatment setting so far [13] – until Sekhar et al. published their work (the “Sekhar Grading System for Cranial Chordomas”; SGSCC) in 2017 classifying patients with SBC into low, intermediate or high-risk groups [14]. As that aforementioned retrospective analysis was performed on a small cohort of only 42 patients, an additional analysis of this score applied on a larger cohort is the goal of this work. Additionally, we also sought to assess which variables predicted for SBC patients’ outcome after pencil beam scanning proton therapy (PBS-PT).

## Methods

### Patients

This retrospective analysis was performed at the center for proton therapy of Paul Scherrer Institute (PSI) in Villigen, Switzerland. Patients (adults and children alike) treated with curative intent for histologically proven SBC with PBS-PT between 1998 and 2016 were included. The presence of metastatic disease was an exclusion criterion for this study. One hundred and ninety-three such patients were identified. Forty-eight (25%) patients were excluded because of lack of pre-operative magnetic resonance imaging (MRI) assessable with full T1/T2 sequences in our Picture Archiving and Communication System. Three (2%) other patients were excluded because they did not consent to the use of their data for publishing purposes. As such, 142 (74%) of the 193 identified patients were eligible for analysis. The patient’s medical records were reviewed for patient characteristics, tumor features, treatment parameters and clinical outcomes. In regards to tumor resection before PBS-PT gross total resection (GTR) was defined as resection of all visible tumor under the surgical microscope and was achieved in 19 (13%) patients. In 118 (83%) patients only sub-total resection (STR) was achieved, but resection adequate to proceed with PBS-PT. Five (4%) patients had a biopsy only. All patient characteristics are detailed in Table 1. This analysis was approved by the Northwest and Central Switzerland Ethics Committee (EKNZ 2018–00621) and conducted in accordance with institutional guidelines and the principles of the Declaration of Helsinki and its subsequent amendments [15].

**Table 1:** Patients’ and disease characteristics (*n* = 142)

Age at diagnosis	median (range) 42 (1–79)
Gender	n (%)
Female	66 (46)
Male	76 (53)
Histology	n (%)
Non-chondroid	136 (96)
Chondroid	6 (4)
Number of surgeries (biopsies included)	n (%)
1	75 (53)
2	46 (32)
> 2	21 (15)
Maximal extent of resection	n (%)
Gross total resection	19 (13)
Subtotal resection	118 (83)
Biopsy	5 (4)
Surgical complications	n (%)
No	88 (62)
Yes	54 (38)
Brainstem compression before PT	n (%)
No	105 (74)
Abuttment	27 (19)
Yes	10 (7)
Optic pathway compression before PT	n (%)
No	121 (85)
Abuttment	15 (11)
Yes	6 (4)
Any compression/abuttment before PT	n (%)
No	88 (62)
Yes	54 (38)
Delivered dose in GyRBE	median (range)
Total dose	74.0 (72.6–80.0)
Single dose	2.0 (1.8–2.5)
GTV in cc	median (range)
	26.3 (0.0–130.4)
PT for recurrent disease	n (%)
No	120 (85)
Yes	22 (15)

*PT* proton therapy, *GyRBE* relative biological effective dose in gray, *GTV* gross tumor volume, *cc* cm^3^

### Sekhar grading system for cranial Chordomas (SGSCC)

For the purpose of this analysis, we computed the SGSCC score according to the paper by Sekhar et al. [14], as a function of tumor size and site, vascular involvement, intradural invasion, and recurrence of the tumor after prior treatment (Table 2). All these variables refer to the state of the SBC directly before the upfront treatment modality (surgery and/or PBS-PT).

**Table 2:** Criteria for the SGSCC

Tumor size (TED)	0–4 points
< 2 cm	1
2 cm to 3.9 cm	2
4 cm to 5.9 cm	3
> 5.9 cm	4
Tumor sites	1 point each
Upper clivus	
Middle clivus	
Lower clivus	
Left petrous bone	
Right petrous bone	
Left cavernous sinus	
Right cavernous sinus	
C1/2/3 left	
C1/2/3 right	
Vascular involvement	1 point each
Left carotid artery	
Right carotid artery	
Basilar artery	
Left vertebral artery	
Right vertebral artery	
Intradural invasion	0–2 points
No	0
Slight	1
With brainstem compression	2
Recurrence after prior treatment	0–5 points
No	0
After surgery	2
After RT	3
After Surgery and RT	5

*TED* tumor equivalent diameter, *C1/2/3* cervical vertebrae 1–3, *RT* radiotherapy

For tumor size we applied the formula for tumor equivalent diameter (TED) D_mean_ = (a*b*c)^1/3^. We scored the 5 criteria as suggested in the original article and sorted the patients according to their score. Scores (ranging from 2 to 25 points) were computed into three prognostic groups (2–7 points low-risk, 8–12 points intermediate-risk and 13–25 points high-risk). Forty three (30%) SBC patients were scored not only by a radiation oncologist but also independently by a neuro-radiologist, so as to assess the inter-variability of the evaluators. The SGSCC scores (median: 10 vs. 9; range: 4–15 vs. 4–16) and grouping were not significantly different between the neuro-radiologist and radiation oncologist (*p = 0.43*). As such, the rest of the cohort was scored by the radiation oncologist only.

### Pencil beam scanning proton therapy

All patients were treated with PBS-PT using the 250 or 590 MeV cyclotrons at PSI, computed with a 3D-treatment planning system (*PSI*-Plan) as described by Weber et al. [9]. Six (4%) patients were also treated with photon therapy to a median dose of 12.0 Gy (range, 2.0–59.4). The median total delivered total dose was 74.0 GyRBE (range, 72.6–80.0). Fractional doses ranged from 1.8 to 2.5 GyRBE with most patients receiving 2.0 GyRBE per fraction. Single-field uniform dose (SFUD) plans and intensity modulated proton therapy (IMPT) plans were used sequentially at PSI.

### Statistical analysis

Time to death and local recurrence (LR) or distant recurrence (DR) were determined from the first day of proton therapy. The first imaging showing LR or DR was the event for local control (LC) and distant control (DC) respectively, whereas death was the event for overall survival (OS). The first imaging showing LR, or patient death due to local tumor progression were the events for local recurrence-free survival (LRFS). Survival rates were calculated using the Kaplan-Meier actuarial method. The log-rank test was used to compare different functions for LC and survival. Two-sided *p*-values were considered statistically significant at *p = < 0.05*. JMP (version 12.2.0; SAS Institute Inc.) was used for statistical analyses.

## Results

After a median follow-up time of 52 months (range, 3–152), 35 (25%) failures were observed. Of those 35 failures, 30 were isolated local recurrences, 4 patients had local and distant failures and one patient presented with an isolated distant failure (Table 3).

**Table 3:** Failures

	n (%)
Only local failure	30 (21)
Only distant failure	1 (1)
Both local and distant failure	4 (3)
Total	35 (25)

At 5 years LC was 75% (95% CI 67–82), and the LRFS was 70% (95% CI 60–78). Twenty-one (14.8%) patients had died during a period of 3 to 95 months (median, 43 months) after PBS-PT, resulting in a 5-year OS of 83% (95% CI 73–88). Cause of death was in 57% of cases (*n* = 12) local tumor progression. Other causes were cardiovascular disease (*n* = 3; 14%), infections (*n* = 2; 10%), second malignancies (n = 3; 14%) and suicide (n = 1; 5%).

Analyzing the patient data according to the SGSCC (Table 4) we found that 8 patients (6%) had tumors smaller than 2 cm, 81 patients (57%) between 2 and 3.9 cm, 51 (36%) between 4 and 5.9 cm and 2 patients (1%) had tumors larger than 6 cm. In 74 cases (52%) the SBC was located in three or fewer sites of the skull base, and 73 patients (51%) showed vascular involvement. Intradural invasion was present in 124 (87%) patients. Only a minority of patients (*n* = 21; 15%) was treated for a recurrence after prior treatment, and just one of them had received radiotherapy before. The low-risk group consisted of 40 patients with a score < 8 (28%), 83 patients with a score of 8 to 12 fell into the intermediate-risk group (59%), and the high-risk group was made up of 19 patients with a score > 12 (13%).

**Table 4:** Patients according to SGSCC

Tumor size (TED)	n (%)
< 2 cm	8 (6)
2 cm to 3.9 cm	81 (57)
4 cm to 5.9 cm	51 (36)
> 5.9 cm	2 (1)
Tumor sites	n (%)
≤ 3	74 (52)
> 3	68 (48)
Vascular involvement	n (%)
≤ 1	73 (51)
> 1	69 (49)
Intradural invasion	n (%)
No	18 (13)
Slight	38 (27)
With brainstem compression	86 (60)
Recurrence after prior treatment	n (%)
No	121 (85)
After surgery	20 (14)
After RT	0 (0)
After Surgery and RT	1 (1)
Total Score	n (%)
< 8	40 (28)
8 to 12	83 (59)
> 12	19 (13)

*SGSCC* Sekhar Grading System for Cranial Chordoma, *TED* tumor equivalent diameter, *RT* radiotherapy

**Fig. 1:**
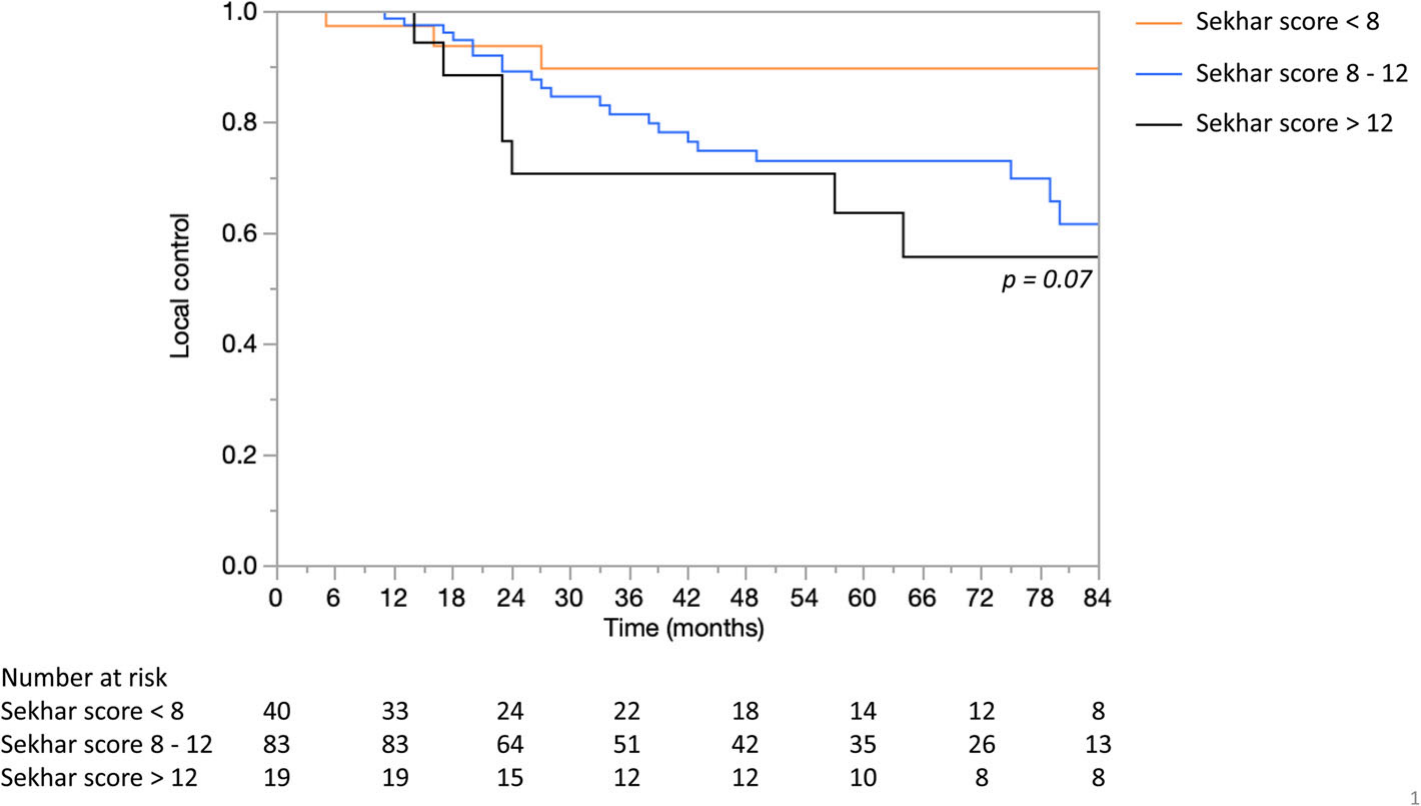
Local control as a function of Sekhar scores for 142 chordoma patients treated with Proton Therapy

At 5 years, we estimated a LC of 90, 72 and 64% (*p = 0.07;* Fig. 1), a LRFS of 93, 89 and 66% (*p = 0.05*) and an OS of 89, 83 and 76% *(p = 0.65)* for the low-, intermediate- and high-risk group, respectively. The difference in LC was significant (*p = 0.04*) after univariable analysis (Table 5), comparing the low-risk group (score of < 8) to the other two (score ≥ 8).

**Table 5:** Univariable Analysis (Log-rank)

	Local recurrence		Overall survival	
	HR (95% CI)	*p*	HR (95% CI)	*p*
Age (years)				
> 42.5 (=median)	0.91 (0.46–1.82)	*0.80*	2.74 (1.11–7.69)	*0.03*
Sekhar score				
< 8	0.31 (0.09–1.03)	*0.04*	1.03 (0.34–2.64)	*0.95*
8–12	1.29 (0.62–2.66)	*0.49*	0.73 (0.31–1.75)	*0.46*
> 12	1.83 (0.82–4.06)	*0.13*	1.57 (0.51–4.04)	*0.37*
Number of sites involved				
> 3 (= median)	0.91 (0.46–1.81)	*0.79*	0.90 (0.38–2.17)	*0.81*
Vascular involvement (number of vessels)				
> 1 (= median)	1.90 (0.54–2.16)	*0.82*	0.80 (0.34–1.92)	*0.61*
≤ 2 vs > 2	1.00 (0.43–2.31)	*0.99*	1.12 (0.37–2.85)	*0.83*
Intradural invasion				
yes vs. no	1.38 (0.42–4.57)	*0.60*	0.75 (0.25–3.20)	*0.64*
PT for recurrent disease				
yes vs. no	3.19 (1.57–6.50)	*< 0.01*	1.92 (0.68–4.73)	*0.17*
Number of oncological surgeries				
> 1 (= median)	1.33 (0.67–2.64)	*0.42*	1.41 (0.60–3.45)	*0.44*
≤ 2 vs > 2	0.38 (0.17–0.85	*0.01*	0.28 (0.12–0.76)	*< 0.01*
Maximum extent of surgery				
GTR vs. STR or Biopsy	1.26 (0.38–4.11)	*0.72*	1.62 (0.26–5.67)	*0.51*
Surgical complications				
yes vs. no	1.90 (0.94–3.81)	*0.07*	2.84 (1.20–6.98)	*0.01*
Gross tumor volume (cc)				
> 26 (= median)	1.26 (0.63–2.50)	*0.51*	2.89 (1.13–8.85)	*0.03*
≤ 40 vs. > 40	2.34 (1.17–4.69)	*0.01*	0.19 (0.07–0.46)	*< 0.01*
Brainstem compression before PT				
yes vs. no	2.53 (1.27–5.01)	*< 0.01*	1.44 (0.55–3.47)	*0.43*
OA compression before PT				
yes vs. no	2.21 (1.02–4.76)	*0.04*	2.14 (0.76–5.29)	*0.11*
Brainstem or OA compression before PT				
yes vs. no	2.46 (1.22–4.96)	*< 0.01*	1.64 (0.69–3.95)	*0.25*

*PT* proton therapy, *GTR* gross total resection, *STR* subtotal resection, *cc* cm^3^, *OA* optic apparatus

In univariable analysis we also looked at individual non-SGSCC prognostic factors. Patients who had received PT for recurrent disease after prior treatment had a significantly worse LC (*p = < 0.01*). Regarding surgical intervention, patients who had been operated on twice or less had not only a better LC (*p = 0.01*) but also a better OS (*p = < 0.01*) than patients with more than two surgeries. There was no significant difference regarding LC or OS when looking at the maximum extent of the surgery (GTR vs. STR or biopsy), whereas surgical complications were an indicator for worse OS (*p = 0.01*).

Patients with > 40 cc of residual disease before PT (measured as gross tumor volume (GTV) used for planning) had a worse LC (*p = 0.01*) and OS (*p = < 0.01*) compared to patients with ≤40 cc. There was also a significant negative impact on LC for patients with tumor compression of the brainstem and/or optic apparatus before PT (*p = < 0.01*) without effect on OS.

## Discussion

### Outcome

Due to their locally aggressive behavior and possible proximity to critical structures such as the brainstem, the prognosis of skull base chordomas patients is mediocre at best with a median survival of 7.7 years described in the literature [1]. In this retrospective analysis the 5-year LC rate was 75% and the 5-year OS was 83%, which is in line with our last publication on chordoma treated with PBS PT [9]. As patients can experience substantial treatment related toxicity, be it after surgery and/or radiotherapy, it is of paramount importance to tailor the treatment intensity with the patient’s prognosis, to optimize the therapeutic ratio. Ideally, one should be able to tailor the adjuvant PT to a SBC patients after surgery, those deemed at high risk of recurrence managed by a dose-escalation paradigm (i.e. > 74 GyRBE) with or without targeted agents and those at lower risk treated with more conventional radiation doses (i.e. ≤ 74 GyRBE) thus decreasing somewhat the likelihood of radiation-induced adverse events [16, 17].

### Prognostic factors

In order to identify patients at higher risk for recurrence many prognostic factors have been described in recent literature, not limited to but including pre-PBS-PT size of tumor, compression of organs at risk (OAR), initial tumor size at diagnosis, the need for a second course of radiation treatment or non-GTR [9–11, 18]. The meta-analysis by Zou et al. [12] gives a very comprehensive overview of many prognostic factors including an array of molecular biomarkers, and whether they are associated with the outcome in patients with SBC. What is currently missing is a comprehensive scoring system, which would include all above-mentioned factors and could be used in the pre-treatment setting – to guide treatment according to the patient’s risk for recurrence but also to manage the tumor surveillance at follow-up. Most local failures occur within 2–3 years after treatment [19]. However, there are some patients who progress or develop recurrences later, as was the case for 7 (5%) patients in this cohort who had a local failure later than 5 years after treatment.

### Previous scoring systems

Before Sekhar et al. devised their SGSCC, only one scoring system had been developed. In 2016, Jun-Peng et al. published the proposal and validation of the “Basic Progression Scoring System for Patients with Skull Base Chordoma” (BPSC) [13]. Retrospectively, 170 patients were separated in a training and validation set. Using the training set, 11 factors were identified that could have an impact on progression. Of those, 5 were significant on multivariate analysis and put in the BPSC – age (< 22 vs. ≥ 22 years), treatment history (previous surgery or radiotherapy), pre-treatment KPS (< 70 vs. ≥ 70), pathology (classical vs. chondroid) and MRI features (ratio of the mean signal intensity of tumor to the mean signal intensity of surrounding brainstem). Based on a total score ranging from 0 to 5 the patients were distributed into three groups, showing a significant difference regarding progression-free survival (PFS). The score was then successfully validated on the second set. To our knowledge, this score never really found its way into clinical practice. Although successfully validated for PFS, it lacks tangible criteria that can actually be taken into account before and during treatment (such as for example tumor size, compression of OAR or vascular involvement). The factors for the BPSC are more general such as age, “Karnofsky Performance Status” (KPS) or previous treatment. Therefore, it gives the health professionals an overall idea of prognosis and can be helpful for follow-up, but does not specifically tell us how to improve and guide treatment itself for an individual SBC patients.

### SGSCC scoring system

As described above, in 2017, Sekhar et al. proposed the SGSCC, a pre-operative scoring system based on a small cohort of 42 patients. Our attempt to assess it using our cohort of 142 patients treated with PBS-PT demonstrated the complexity of factors affecting the outcome of patients with this tumor. On univariable analysis there was a significant benefit regarding LC for patients in the low-risk group compared to the other two groups (Fig. 1), suggesting its clinical value for the management of SBC patients, be it before or after diagnosis. The lack of statistical significance for the three groups could be due to the small sample size of 142 patients for this rare skull-base tumor.

That said we have to understand that both analyses looked at the problem from two different perspectives - the Sekhar group from a surgical and we from a radio-oncological point of view. Comparing the two cohorts, we realized they differed in some aspects (Table 6). In our cohort, there were fewer large tumors, less patients were treated for recurrent disease and GTR was achieved in fewer cases. Size of GTV was not recorded in the original paper, but initial tumor size was very similar in the two cohorts (TED of 3.3 vs. 3.4 cm). In both, there were many tumors showing intradural invasion with a comparable number of sites involved. The age of the patients in the Sekhar et al. paper and our cohort was almost identical.

**Table 6:** Comparison of the two cohorts

	This study (*n* = 142)	Sekhar (*n* = 42)
Age (years)	42 (median)	41 (mean)
GTR (%)	13	36
Follow-up (months)	51	50
Local recurrence (%)	24	19
LC (%)	75 (at 5 y)	81 (at 1 y)
OS (%)	83 (at 5 y)	100 (at 1 y)
SGSCC Score		
< 8 (%)	28	19
8 to 12 (%)	59	55
> 12 (%)	13	26
TED (cm)	3.4 (median)	3.3 (mean)
Number of sites involved	3 (median)	4.6 (mean)
Vascular involvement (number of vessels)	1 (median)	na
Intradural invasion (%)	87	73
Treatment for recurrence (%)	15	40

*GTR* gross total resection, *LC* local control, *OS* overall survival, *SGSCC* Sekhar Grading System for Cranial Chordoma, *TED* tumor equivalent diameter

It is thus questionable if these differences could account for the lack of statistical significance in our analysis. To see if our cohort included any outliers we also compared it to other cohorts, such as the Centre de protonthérapie Orsay-Curie [20] and Heidelberg patients [21] as well as our older cohort from the publication in 2016 [9]. We did not observe major differences between the various institutional cohorts (Table 7).

**Table 7:** Comparison with older cohorts

	This study (*n* = 142)	Weber 2016 (*n* = 151)	Paris D’Orsay (*n* = 34)	Heidelberg (*n* = 155)
Age (years)	42	43 (mean)	59	48
GTV (cc)	26	35	24	70 (PTV)
Treatment for recurrence (%)	15	36	44	35
GTR (%)	13	4	9	0
LC (%)	75 (at 5 y)	76 (at 5 y)	83 (at 3 y)	72 (at 5 y)
OS (%)	83 (at 5 y)	86 (at 5 y)	91 (at 3 y)	85 (at 5 y)

*GTV (cc)* gross tumor volume in cm^3^, *GTR* gross total resection, *LC* local control, *OS* overall survival

In our analysis, the most significant risk factors for LC seem to be recurrence after prior treatment (surgery or radiotherapy), the number of oncological surgeries (more than two) as well as the extent of surgery in regards to GTV (> 40 cc) and proximity to OAR (compression of brain stem and optic apparatus) before PBS-PT. There was a significant difference in LC and/or OS after univariable analysis, which could make them useful for treatment guidance. Recurrence after prior treatment was already included in the SGSCC, whereas the other three were not, since it is a preoperative grading system. The question remains if a postoperative prognostic scoring system including the above-mentioned prognostic factors could be of clinical value. This is particularly relevant because SBC patients should be managed by surgery and adjuvant radiation therapy and a need for a holistic scoring system, not limited to the pre-operative setting is urgently needed.

The main limitations of this analysis are its retrospective nature and the fact that we looked at our cohort not in the preoperative setting but after surgery before adjuvant PBS-PT. As a result, some endpoints of the original paper could not be included in our analysis. Additionally, the scoring of the whole study cohort was not performed by a neuroradiologist but by a radiation oncologist. Having said that, these data suggest that scoring can be indeed performed by a radiation oncologist managing routinely SBC patients.

## Conclusions

Our assessment of the SGSCC for our patients treated with PBS-PT showed a trend towards better outcome for patients with lower scores as compared to those with higher scores. This is probably an indicator that the scoring system could be useful to guide treatment and patient follow-up for SBC patients. However, this SGSCC score can only be applied in the pre-operative and not post-operative setting as advocated by the authors. It remains to be determined if a postoperative scoring system, not limited to but including compression of the OARs, pre-PBS-PT tumor volume, should be computed.

## Data Availability

The datasets used and/or analyzed during the current study are available from the corresponding author on reasonable request.

## Abbreviations

SBC: Skull-base Chordoma
SGSCC: Sekhar Grading System for Cranial Chordomas
PBS-PT: Pencil beam scanning-proton therapy
PSI: Paul Scherrer Institute
MRI: Magnetic resonance imaging
GTR: Gross total resection
STR: Subtotal resection
EKNZ: Ethikkommission Nordwest- und Zentralschweiz
TED: Tumor equivalent diameter
Gy: Gray
GyRBE: Gray-relative biological effectiveness
SFUD: Single-field uniform dose
IMPT: Intensity modulated proton therapy
LR: Local recurrence
DR: Distant recurrence
LC: Local control
DC: Distant control
OS: Overall survival
LRFS: Local recurrence-free survival
GTV: Gross tumor volume
OAR: Organ at risk
BPSC: Basic Progression Scoring System for Patients with Skull Base Chordoma
PFS: Progression-free survival
KPS: Karnofsky Prognostic score

## References

[1] Smoll NR, Gautschi OP, Radovanovic I, Schaller K, Weber DC (2013). Incidence and relative survival of chordomas: the standardized mortality ratio and the impact of chordomas on a population. Cancer.

[2] Healey JH, Lane JM (1989). Chordoma: a critical review of diagnosis and treatment. Orthop Clin North Am.

[3] McMaster ML, Goldstein AM, Bromley CM, Ishibe N, Parry DM (2001). Chordoma: incidence and survival patterns in the United States, 1973-1995. Cancer Causes Control.

[4] Chugh R, Tawbi H, Lucas DR, Biermann JS, Schuetze SM, Baker LH (2007). Chordoma: the non-sarcoma primary bone tumor. Oncologist.

[5] Kayani B, Sewell MD, Tan KA, Hanna SA, Williams R, Pollock R (2015). Prognostic factors in the operative management of sacral chordomas. World Neurosurg.

[6] Di Maio S, Temkin N, Ramanathan D, Sekhar LN (2011). Current comprehensive management of cranial base chordomas: 10-year meta.Analysis of observational studies. J Neurosurg.

[7] Fuchs B, Dickey ID, Yaszemski MJ, Inwards CY, Sim FH (2005). Operative management of sacral chordoma. J Bone Joint Surg Am.

[8] Tzortzidis F, Elahi F, Wright DC, Temkin N, Natarajan SK, Sekhar LN (2006). Patient outcome at long-term follow-up after aggressive microsurgical resection of cranial base chordomas. Neurosurgery.

[9] Weber DC (2016). Long term outcomes of patients with skull-base low-grade chondrosarcoma and chordoma patients treated with pencil beam scanning proton therapy. Radiother Oncol.

[10] Arman J (2015). Factors predicting recurrence after resection of Clival Chordoma using variable surgical approaches and radiation modalities. Neurodurgery.

[11] Bohmann LE (2014). Skull Base Chordoma and Chondrosarcoma: influence of clinical and demographic factors on prognosis: a SEER analysis. World Neurosurg.

[12] Zou MX (2018). Prognostic factors in Skull Base Chordoma: a systematic literature review and meta-analysis. World Neurosurg.

[13] Jun-Peng M (2016). Proposal and validation of a basic progression scoring system for patients with Skull Base Chordoma. World Neurosurg.

[14] Brito da Silva H, Straus D, Barber JK, Rostomily R, Ferreira M, Sekhar LN. Cranial Chordoma: a new preoperative grading system. Neurosurgery 2017;0:1–13. 10.1093/neuros/nyx423 PMC6140779 29126120

[15] World Medical Association Declaration of Helsinki: ethical principles for medical research involving human subjects. JAMA. 2013;310(20):2191–4. 10.1001/jama.2013.281053 24141714

[16] Hindi N (2015). Imatinib in advanced chordoma: a retrospective case series analysis. Eur J Cancer.

[17] Magnaghi P (2018). Afatinib is a new therapeutic approach in Chordoma with a unique ability to target EGFR and Brachyury. Mol Cancer Ther.

[18] Sanusi O, Arnaout O, Rahme RJ, Horbinski C, Chandler JP. Surgical Resection and Adjuvant Radiation Therapy in the Treatment of Skull Base Chordomas. World Neurosurg 2018;115:e13–21. 10.1016/j.wneu.2018.02.127 29545225

[19] Yasuda M, Bresson D, Chibbaro S, Cornelius JF, Polivka M, Feuvret L (2012). Chordomas of the skull base and cervical spine: clinical outcomes associated with multimodal surgical resection combined with proton-beam radiation in 40 patients. Neurosurg Rev.

[20] Georges N (2001). Combination of photon and proton radiation therapy for Chordomas and Chondrosarcomas of the Skull Base: the Centre de Protonthérapie D’Orsay experience. Int J Radiation Oncology Biol Phys.

[21] Uhl M (2014). Highly effective treatment of Skull Base Chordoma with carbon ion irradiation using a raster scan technique in 155 patients: first long-term results. Cancer.

